# Security and Reliability Analysis of the Power Splitting-Based Relaying in Wireless Sensors Network

**DOI:** 10.3390/s24041300

**Published:** 2024-02-17

**Authors:** Minh Tran, Lam-Thanh Tu, Bui Vu Minh, Quang-Sang Nguyen, Lubos Rejfek, Byung Moo Lee

**Affiliations:** 1 Communication and Signal Processing Research Group, Faculty of Electrical and Electronics Engineering, Ton Duc Thang University, Ho Chi Minh City 70000, Vietnam; tranhoangquangminh@tdtu.edu.vn (M.T.); tulamthanh@tdtu.edu.vn (L.-T.T.); 2 Faculty of Engineering and Technology, Nguyen Tat Thanh University, 300A-Nguyen Tat Thanh, Ward 13, District 4, Ho Chi Minh City 70000, Vietnam; bvminh@ntt.edu.vn; 3 Science and Technology Application for Sustainable Development Research Group, Ho Chi Minh City University of Transport, Ho Chi Minh City 70000, Vietnam; sang.nguyen@ut.edu.vn; 4 Faculty of Electrical Engineering and Informatics, University of Pardubice, 53210 Pardubice, Czech Republic; lubos.rejfek@upce.cz; 5 Department of Intelligent Mechatronics Engineering, and Convergence Engineering for Intelligent Drone, Sejong University, Seoul 05006, Republic of Korea

**Keywords:** intercept probability, Internet of Things, outage probability, performance analysis, relaying networks

## Abstract

This paper studies the security and reliability of the power splitting (PS)-based relaying in the Internet of Things (IoT) networks with the help of a jammer. Based on the considered system model, we derive outage probability (OP) and intercept probability (IP) under two distinguished schemes, namely, the static PS relaying (SPSR) scheme and the dynamic PS relaying (DPSR) scheme. More precisely, the PS ratio of the former is a constant number, while the latter is optimally adjusted in order to minimize the OP and counts only on the channel gain of the second hop. Numerical results are provided to not only verify the accuracy of the proposed mathematical framework but also identify the trends of both OP and IP with respect to several important parameters. Our findings unveil that the OP and IP have contradictory behavior with respect to the transmit power and number of sources. Moreover, the performance of the DPSR scheme is superior to that of the SPSR scheme.

## 1. Introduction

It is expected that there will be 28.5 billion devices connected to the internet for realizing the Internet of Things (IoT) [1,2]. However, one of the principal challenges of such networks is how to provide energy to supply these devices in order to allow them to continuously operate over a reasonable amount of time and not make their battery and/or devices too bulky. Additionally, some devices are located in remote areas; thus, constantly charging these devices is almost infeasible [3].

Under this context, a recent emerging technology called simultaneous wireless information and power transfer (SWIPT) constitutes a suitable solution to not only prolong the battery life but also guarantee the high quality of services (QoSs) [4,5,6,7]. Particularly, SWIPT is an advanced technology that allows the carrier frequency concurrently bearing information and replenishing the receiver’s battery. To realize the SWIPT technology, three main protocols are proposed in the literature: namely, time-switching, power-splitting, and antenna-switching protocols. The first and second approaches split the radio frequency in the time and power domain, while the last one splits in the spatial domain. More precisely, the received power is put into the energy harvester for the portion of the whole transmission duration and is put into the information decoder for the remaining transmission duration in the time-switching protocol. The power-splitting protocol, on the other hand, directly splits the received power into two parts: one puts it into the energy harvester and another sends it to the information decoder [8]. Regarding the antenna-switching protocol, some antennae are used for harvesting energy and others are used for decoding information [9,10,11,12].

Physical layer security (PLS), on the other hand, is a technology that improves information security along with the conventional approach utilizing high complexity cryptography techniques [13,14,15]. More precisely, PLS exploits the different characteristics of the channels between the legitimate and the eavesdropper. Wyner in [16] proved that one can attain perfect security provided that the quality of the legitimate link is superior to the eavesdropper link. To enhance the performance of PLS, many technologies have been proposed in the literature, and one of the promising solutions is to employ a relaying technique which is a mature technology and is proven as an effective solution in improving diversity gain, extending the coverage area.

### State of the Art

The performance of SWIPT-assisted networks and PLS was studied in [17,18,19,20,21,22,23,24,25]. Particularly, the coverage probability of closed-loop multiple-input multiple-output (MIMO) SWIPT-enabled cellular networks was studied in [17]. The results unveiled that both large-scale antenna arrays and ultra-dense deployments of base stations (BSs) are necessary to harvest an amount of power of the order of a milliwatt. The work in [18], on the other hand, investigated the receiver diversity of the cellular networks with SWIPT. The outage probability (OP) and symbol error rate of the two-way energy-harvesting (EH) relaying networks over Rician fading channels were computed in [19,20]. More precisely, these metrics are derived under two transmission schemes, i.e., the delay-limited and delay-tolerant, respectively. This work, however, ignores the direct communications between two source nodes. The work in [21] investigated the OP of the cognitive radio networks (CRNs) considering the random location of the relay nodes. However, the OP was computed based on numerical results rather than in closed-form expression. Thus, it is hard to gain insights from the mathematical framework. The OP of the wireless information and power transfer overlay CRNs networks was evaluated in [22] by employing a numerical method. The secrecy performance of the conventional and CRNs was studied in [23,24]. To be more precise, the secrecy OP (SOP) performance of a single source and multiple legitimate users and eavesdroppers was investigated in [23]. Nonetheless, this work did not consider the assistance of both jammer and relay to effectively enhance information security. Singh et al. in [24] derived the SOP and the intercept probability (IP) of the CRNs with single or multiple primary users. However, the help of the relay was not taken into consideration in this work. The study conducted in [25] delves into the performance of vehicular edge computing systems utilizing non-orthogonal multiple access (NOMA). It jointly optimizes cluster selection, transmit power, and computation resource allocation, demonstrating superiority over various benchmarks. However, their emphasis is on vehicle communications with NOMA, whereas our work centers around wireless sensor networks. Furthermore, our investigation encompasses not only security considerations but also emphasizes reliability.

Although the performance of SWIPT-enabled networks as well as the PLS was well studied as provided above, the performance of SWIPT-assisted relaying networks is still in the infancy stage. Particularly, only a few works have investigated such an interesting problem [26,27,28,29,30,31]. For example, the work in [26] investigated both the security and reliability of the two-way cognitive relaying networks. More precisely, the relay which had the highest end-to-end (e2e) signal-to-noise ratio (SNR) was selected to help with exchanging confidential information between two sources where the direct link was omitted due to the deep fades. Despite a significant simplification in the mathematical framework, this scenario is not always maintained in practice. The authors in [27] also addressed the security and reliability of wireless-powered relaying networks under the impact of I/Q imbalance. Specifically, the authors derived the OP and IP, where the channel estimation errors along with the in-phase and quadrature-phase imbalance were taken into consideration. The asymptotic framework under a high SNR regime was provided as well. The work in [28], on the other hand, addressed the secrecy performance of the SWIPT-enabled underlay CRNs. Particularly, the authors derived the SOP of the desired user. Nonetheless, this work does not take the support of either relay or jammer into consideration to enhance the system performance. The authors in [30] also investigated security and reliability issues in wireless sensor networks. Despite their exploration of multi-source wireless sensor networks, they did not incorporate a jammer to actively reduce the intercept probability. Moreover, their use of an AF-based relay differs from our approach, where we employ a DF-based relay, which is known to offer superior performance. The investigation of the security and reliability trade-off in SWIPT-enabled full-duplex relaying NOMA networks was explored in the literature. Specifically, in the work conducted by Nguyen et al. [31], the focus was on the security and reliability aspects within NOMA networks employing SWIPT. The authors in [32] derived OP and IP metrics for multi-hop relaying systems incorporating reconfigurable intelligent surfaces (RISs). However, the utilization of friendly jamming techniques was not incorporated into the framework in their work.

In this work, different from the above-mentioned works, we analyze the reliability and security of the SWIPT-based relaying networks in order to highlight the benefits of the combination of SWIPT and relaying technology in wireless networks. More precisely, we derive the OP and IP of multiple sources power splitting (PS)-based relaying networks with the help of a jammer. The main contributions and novelties are reported below:

- We propose and comprehensively investigate the benefits of two PS-based relaying schemes: (i) static power splitting-based relaying (SPSR) and (ii) dynamic power splitting-based relaying (DPSR).
- We consider the opportunistic source transmission in place of joint transmission to enhance the system diversity gain, reduce the synchronization complexity, and limit the interception probability.
- To the best of the authors’ knowledge, this paper is a pioneer in deriving the closed-form expressions of the OP and IP under the multiple sources PS-based relaying networks with the assistance of the jammer. The considered problem is challenging as it involves many random variables that are not independent of each other, thereby complicating derivation.
- We provide the closed-form expressions of OP and IP of the fixed and optimal power-splitting ratio in the SPSR and DPSR schemes, respectively.
- The accuracy of the derived mathematical framework is verified via Monte Carlo simulations. We show that there is contrary behavior of OP and IP regarding the transmit power of source nodes and the number of sources. Moreover, our findings also illustrate that the DPSR scheme outperforms the others in terms of the OP, but this is not the case for the IP.

The rest of this paper is organized as follows. The system model is given in Section 2. The derivation of key performance metrics, including the OP and IP of the proposed system, is provided in Section 3. Numerical results are presented in Section 4. Section 5 concludes the paper.

## 2. System Model

**Figure 1:**
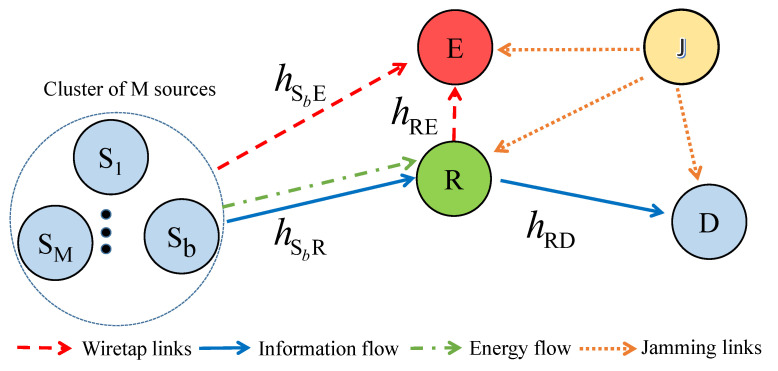
System model.

Let us consider a wireless network comprising M source nodes ($S_{1},\ldots ,S_{M}$) to transmit information to the destination (D) with the assistance of a decode-and-forward (DF) relay (R), as shown in Figure 1. Additionally, there is a passive eavesdropper (E) attempting to wiretap confidential information from S to D and an active jammer (J) who assists in the exchange of information from S to D. All nodes are equipped with a single antenna (the extension to multiple antennas at the source, relay, destination, and eavesdropper can be straightforwardly applied by employing maximal ratio combining, maximal ratio transmission, transmit antenna selection, and selection combining at the transmitter and/or receiver, as discussed in [33]).

### 2.1. Channel Modeling

Denote $h_{\mathrm{XY}}$, $\mathrm{XY}\in \left(S_{o}E,\;S_{o}R,\;\mathrm{RE},\;\mathrm{RD},\;\mathrm{JE},\;\mathrm{JR},\;\mathrm{JD}\right)$, $o\in \left\{1,\ldots ,M\right\}$, as the channel coefficients of the transmission link from X to Y followed by Rayleigh distribution. The channel gain denoted by $\gamma_{\mathrm{XY}}={\left|h_{\mathrm{XY}}\right|}^{2}$, as a result, follows an exponential distribution whose cumulative distribution function (CDF) and probability density function (PDF) are given as follows [34]:

(1) $$\begin{array}{cc} F_{\gamma_{\mathrm{XY}}}\left(x\right)= & 1-\exp(-\lambda_{\mathrm{XY}}x),\\ f_{\gamma_{\mathrm{XY}}}\left(x\right)= & \frac{\partial F_{\gamma_{\mathrm{XY}}}\left(x\right)}{\partial x}=\lambda_{\mathrm{XY}}\exp\left(-\lambda_{\mathrm{XY}}x\right), \end{array}$$

where $\lambda_{\mathrm{XY}}$ represents the large-scale path loss from X to Y and is formulated as

(2) $$\begin{array}{c} \lambda_{\mathrm{XY}}={\left(d_{\mathrm{XY}}\right)}^{\beta }, \end{array}$$

where $d_{\mathrm{XY}}$ is the transmission distance between X and Y and β is the path-loss exponent. $F_{X}\left(x\right)$ and $f_{X}\left(x\right)$ denote the CDF and PDF of RV *X*. Block fading is considered in which channel coefficients are stable for the whole transmission *T* and change independently across different transmissions.

### 2.2. Power Splitting (PS)-Based Relaying

Considering the power-splitting protocol, the received power at R is then split into two parts according to the power-splitting ratio denoted by ρ, 0≤ρ≤1, where the first part is fed into the energy-harvested receiver and the remaining part is used for the information receiver. Additionally, we consider two distinct PS schemes, the static and dynamic PS scheme. The ρ of the former one is a constant number, while the latter is optimally adjusted to maximize the end-to-end signal-to-noise ratios (SNRs) at the desired receiver D, thus minimizing the OP.

### 2.3. Transmission Procedure

**Figure 2:**
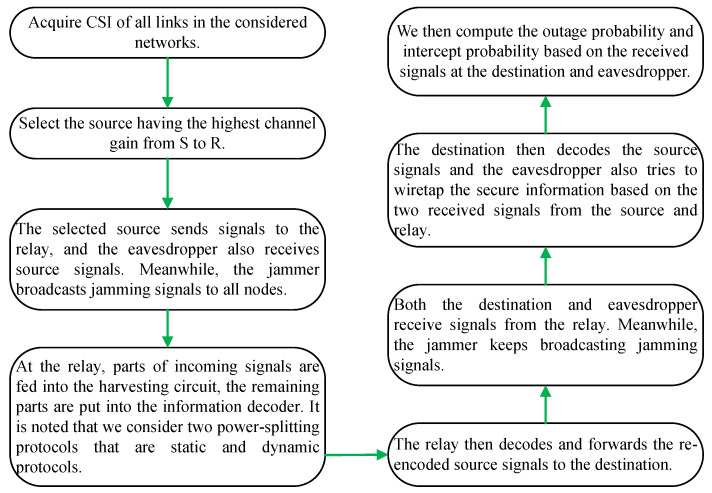
The simplified network scheme of the considered networks.

The whole transmission takes place in two phases. In the first phase, R receives signals from the $S_{n}$, which has the highest channel gain to R, i.e., $\gamma_{S_{n}R}=\max_{o\in \left\{1,\ldots ,M\right\}}\left\{\gamma_{S_{o}R}\right\}$. It is noted that we adopt the opportunistic source transmission instead of employing joint transmission [35] in order to ameliorate the system diversity gain and significantly reduce the synchronization complexity. Meanwhile, the jammer J broadcasts jamming signals in order to prevent eavesdropper E from wiretapping the legitimate information. The received signals at R and E denoted by $y_{R}$ and $y_{E}^{1}$ are then formulated as follows:

(3) $$\begin{array}{cc} y_{R}= & \sqrt{1-\rho }\sqrt{P_{S_{n}}}h_{S_{n}R}x_{S_{n}}+\sqrt{P_{J}}h_{\mathrm{JR}}x_{J}+n_{R},\\ y_{E}^{1}= & \sqrt{P_{S_{n}}}h_{S_{n}E}x_{S_{n}}+\sqrt{P_{J}}h_{\mathrm{JE}}x_{J}+n_{E}, \end{array}$$

where $x_{S_{n}}$ and $x_{J}$ with $E\left\{{\left|x_{S_{n}}\right|}^{2}\right\}=E\left\{{\left|x_{J}\right|}^{2}\right\}=1$ are the transmitted symbol from $S_{n}$ and J, respectively; $E\left[\cdot \right]$ denotes the expectation operator; $P_{S_{n}}=P_{S},\forall n$, $P_{J}$ represents the transmit power of S_*n*_ and J; $n_{R}$, $n_{E}$ are the zero mean additive white Gaussian noise (AWGN) with variance $N_{0}$. The main notations and mathematical symbols are presented in Table 1. The whole transmission procedure of the considered networks is shown in Figure 2.

Moreover, we consider the friendly jammer which is merely against the eavesdropper. Hence, relay R has advanced information to effortlessly remove the jamming signals from its received signal. The received signal at R is then rewritten as

(4) $$\begin{array}{c} y_{R}=\sqrt{1-\rho }\sqrt{P_{S_{n}}}h_{S_{n}R}x_{S_{n}}+n_{R}. \end{array}$$

**Figure 3:**
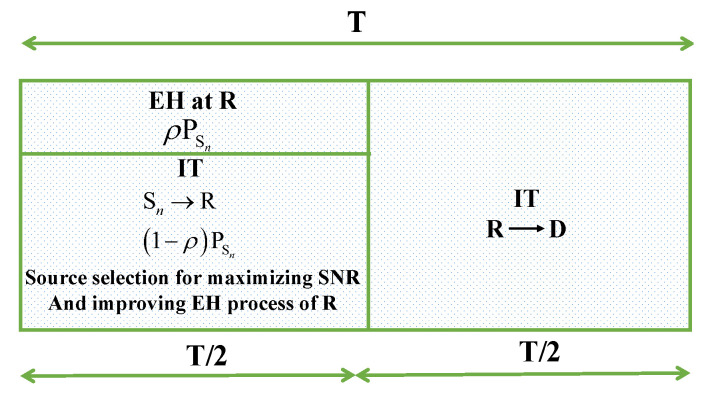
Schematic illustration of EH and IT processes with power-splitting protocol at the relay.

At the end of the first phase, relay R decodes, re-encodes, and forwards the source signals to the destination in the second phase. The whole transmission procedure of the legitimate link is shown in Figure 3. The received signals at D are formulated as

(5) $$\begin{array}{c} y_{D}=\sqrt{P_{R}}h_{\mathrm{RD}}x_{R}+n_{D}, \end{array}$$

where $n_{D}$ is the AWGN noise at D with zero mean and variance $N_{0}$; $x_{R}$ represents the signals sent by R with $E\left\{{\left|x_{R}\right|}^{2}\right\}=1$. It is noted that in (5), we already suppress the jamming signals from J in the second phase. Here, $P_{R}$ is the transmit power of relay R and is based on the amount of harvested energy in the first phase [29]:

(6) $$\begin{array}{c} P_{R}=\eta \rho P_{S_{n}}\gamma_{S_{n}R}, \end{array}$$

where 0<η≤1 is the energy conversion efficiency that takes into account the energy loss owing to the harvesting and decoding circuits. Additionally, jammer J keeps sending the same signals in the second phase to the eavesdropper. The received signals at E in the second phase denoted by $y_{E}^{2}$ are then given by

(7) $$\begin{array}{c} y_{E}^{2}=\sqrt{P_{R}}h_{\mathrm{RE}}x_{R}+\sqrt{P_{J}}h_{\mathrm{JE}}x_{J}+n_{E}^{2}. \end{array}$$

**Figure 4:**
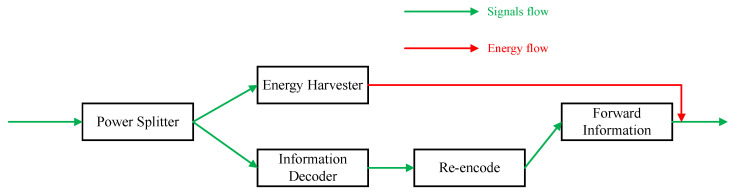
The signals and energy flow at the relay.

Since the DF protocol is considered, the SNR at D ($\gamma_{e2e}$) and the instantaneous rate ($C_{D}$) are formulated as, respectively,

(8) $$\begin{array}{c} \gamma_{e2e}=\min\left(\gamma_{R},\gamma_{D}\right), \end{array}$$

(9) $$\begin{array}{c} C_{D}=\frac{1}{2}\log_{2}\left(1+\gamma_{e2e}\right), \end{array}$$

where $\gamma_{R}$, $\gamma_{D}$ are the SNR of the first phase at R and of the second phase at D and are given as

(10) $$\begin{array}{cc} \gamma_{R}= & \frac{\left(1-\rho \right)\gamma_{S_{n}R}P_{S}}{N_{0}}=\left(1-\rho \right)\gamma_{S_{n}R}\Psi ,\\ \gamma_{D}= & \eta \rho \Psi \gamma_{S_{n}R}\gamma_{\mathrm{RD}}, \end{array}$$

where $\Psi =\frac{P_{S}}{N_{0}}$. The energy and signal flows through the relay are given in Figure 4.

The eavesdropper, on the other hand, employs a selection combining (SC) technique to combine the received signals of two phases in order to intercept the legitimate link. Since the passive eavesdropper is considered, thus, the eavesdropper E does not have full channel-state information (CSI) from S and R, so the SC technique is employed in place of the maximal ratio combining (MRC) technique.

Mathematically speaking, the e2e SNR at E denoted by $\gamma_{E}^{e2e}$ is given as

(11) $$\begin{array}{cc} \gamma_{E}^{e2e}= & \max\left(\gamma_{E}^{1},\gamma_{E}^{2}\right). \end{array}$$

Here, $\gamma_{E}^{1}$, $\gamma_{E}^{2}$ are the SNR of the first and second phase at E and are given as

(12) $$\begin{array}{cc} \gamma_{E}^{1}= & \frac{\gamma_{S_{n}E}\Psi }{\Phi \gamma_{\mathrm{JE}}+1}\approx \frac{\gamma_{S_{n}E}\Psi }{\Phi \gamma_{\mathrm{JE}}},\\ \gamma_{E}^{2}= & \frac{\eta \rho \Psi \gamma_{S_{n}R}\gamma_{\mathrm{RE}}}{\Phi \gamma_{\mathrm{JE}}+1}\approx \frac{\eta \rho \Psi \gamma_{S_{n}R}\gamma_{\mathrm{RE}}}{\Phi \gamma_{\mathrm{JE}}}, \end{array}$$

where $\Phi =\frac{P_{J}}{N_{0}}$.

The instantaneous rate at node E is formulated as

(13) $$\begin{array}{c} C_{E}=\frac{1}{2}\log_{2}\left(1+\gamma_{E}^{e2e}\right). \end{array}$$

Having obtained the e2e SNRs at both D and E, we investigate two important metrics, the OP and IP of the considered system in the next section.

## 3. Performance Analysis

In the present work, we address the OP at the destination and the IP at the eavesdropper under two distinguished power-splitting relaying protocols, i.e., SPSR and DPSR. More precisely, the former investigates the system performance in which the power-splitting ratio is a constant number while the latter aims to maximize the system capacity by optimally adjusting the PS ratio. Following Lemma 1 is useful to compute these metrics.

**Lemma** **1.**  *Let there be a set of independent and identically distributed (i.i.d.) exponential random variables (RVs) with parameters λ denoted by $X_{m},m\in \left\{1,\ldots ,M\right\}$. The CDF and PDF of the maximal RV denoted by $X_{\max}=\max_{m\in \left\{1,\ldots ,M\right\}}\left\{X_{m}\right\}$ are given as follows:*

(14) $$\begin{array}{cc} F_{X_{\max}}\left(x\right)= & 1+\sum_{m=1}^{M}{\left(-1\right)}^{m}C_{M}^{m}\exp\left(-m\lambda x\right)\\ f_{X_{\max}}\left(x\right)= & \lambda \sum_{m=1}^{M-1}{\left(-1\right)}^{m}C_{M-1}^{m}\exp\left(-\left(m+1\right)\lambda x\right) \end{array}$$

*where $C_{M}^{k}=\frac{M!}{k!(M-k)!}$ is the binomial coefficient.*

**Proof.**  The proof is available in Appendix A.  □

### 3.1. Static Power Splitting-Based Relaying (SPSR)

The OP at D and IP at E of the SPSR scheme are presented in this section.

#### 3.1.1. Outage Probability Analysis

OP refers to the probability that the instantaneous SNR is below a predefined threshold. The OP of the SPSR scheme denoted by ${\mathrm{OP}}_{\mathrm{SPSR}}$ is calculated as

(15) $$\begin{array}{cc} \; & {\mathrm{OP}}_{\mathrm{SPSR}}\left(C_{\mathrm{th}}\right)=1+\sum_{k=1}^{M}{\left(-1\right)}^{k}C_{M}^{k}\exp\left(-\lambda_{\mathrm{RD}}\xi -k\lambda_{\mathrm{SR}}\vartheta \right)\\ \; & +\sum_{k=1}^{M}\sum_{m=1}^{N}\frac{\pi {\left(-1\right)}^{k}C_{M}^{k}\lambda_{\mathrm{RD}}\xi }{2N}\sqrt{1-\mu_{m}^{2}}\exp\left(-\frac{\lambda_{\mathrm{RD}}\xi }{2}-\frac{k\lambda_{\mathrm{SR}}\gamma_{th}}{\eta \rho \Psi \Xi (\theta_{m})}-\frac{\lambda_{\mathrm{RD}}\xi \theta_{m}}{2}\right). \end{array}$$

where $\xi =\frac{\left(1-\rho \right)}{\eta \rho }$, $\vartheta =\frac{\gamma_{th}}{(1-\rho )\Psi }$, $\mu_{m}=\cos\left(\frac{\pi (2m-1)}{2M}\right)$; $\theta_{m}=\frac{\xi }{2}\mu_{m}+\frac{\xi }{2}$, $\gamma_{\mathrm{th}}=2^{2C_{\mathrm{th}}}-1$; $C_{\mathrm{th}}$ is the targeted capacity (in bps/Hz) and *N* is a control parameter of the Gaussian–Chebyshev quadrature approximation [36].

**Proof.**  Let us begin the proof with the definition of the OP as follows:

(16) $$\begin{array}{cc} \; & {\mathrm{OP}}_{\mathrm{SPSR}}\left(C_{\mathrm{th}}\right)=\mathrm{Pr}\left(C_{D}=\min\left(\gamma_{R},\gamma_{D}\right)<C_{\mathrm{th}}\right)\\ \; & =\mathrm{Pr}\left(\min\left(\left(1-\rho \right)\gamma_{S_{n}R}\Psi ,\eta \rho \Psi \gamma_{S_{n}R}\gamma_{\mathrm{RD}}\right)<\gamma_{\mathrm{th}}\right)\\ \; & =1-\mathrm{Pr}\left(\gamma_{S_{n}R}\geq \frac{\gamma_{th}}{(1-\rho )\Psi },\gamma_{S_{n}R}\gamma_{\mathrm{RD}}\geq \frac{\gamma_{th}}{\eta \rho \Psi }\right)\\ \; & =1-\int_{0}^{\xi }f_{\gamma_{\mathrm{RD}}}\left(y\right)dy\int_{\frac{\gamma_{th}}{\eta \rho \Psi y}}^{\infty }f_{\gamma_{S_{n}R}}\left(x\right)dx-\int_{\xi }^{\infty }f_{\gamma_{\mathrm{RD}}}\left(y\right)dy\int_{\vartheta }^{\infty }f_{\gamma_{S_{n}R}}\left(x\right)dx, \end{array}$$

where $\xi =\frac{\left(1-\rho \right)}{\eta \rho }$, $\vartheta =\frac{\gamma_{th}}{(1-\rho )\Psi }$ and $\gamma_{\mathrm{th}}=2^{2C_{\mathrm{th}}}-1$. With the help of Lemma 1, (16) is rewritten as follows:

(17) $$\begin{array}{cc} \; & OP_{\mathrm{SPSR}}\left(C_{\mathrm{th}}\right)=1+\sum_{k=1}^{M}{\left(-1\right)}^{k}C_{M}^{k}e^{-\lambda_{\mathrm{RD}}\xi -k\lambda_{\mathrm{SR}}\vartheta }\\ \; & +\sum_{k=1}^{M}{\left(-1\right)}^{k}C_{M}^{k}\lambda_{\mathrm{RD}}\int_{0}^{\xi }\exp\left(-\frac{k\lambda_{\mathrm{SR}}\gamma_{th}}{\eta \rho \Psi y}-\lambda_{\mathrm{RD}}y\right)dy,\\ \; & \overset{\left(a\right)}{=}1+\sum_{k=1}^{M}{\left(-1\right)}^{k}C_{M}^{k}\exp\left(-\lambda_{\mathrm{RD}}\xi -k\lambda_{\mathrm{SR}}\vartheta \right)\\ \; & +\sum_{k=1}^{M}\frac{{\left(-1\right)}^{k}C_{M}^{k}\lambda_{\mathrm{RD}}\xi }{2}\exp\left(-\frac{\lambda_{\mathrm{RD}}\xi }{2}\right)\int_{-1}^{1}\exp\left(-\frac{k\lambda_{\mathrm{SR}}\gamma_{th}}{\eta \rho \Psi \Xi (x)}-\frac{\lambda_{\mathrm{RD}}\xi x}{2}\right)dx, \end{array}$$

where $\left(a\right)$ is held by changing the variable $y=\frac{\xi }{2}x+\frac{\xi }{2}$ and $\Xi \left(x\right)=\frac{\xi }{2}x+\frac{\xi }{2}$. Inspecting the integration in (17), unfortunately, it can compute in closed-form expression owing to the generality of the integration limits. As a consequence, we propose employing the Gaussian–Chebyshev quadrature approximation [36]; the OP is then computed as follows:

(18) $$\begin{array}{cc} \; & {\mathrm{OP}}_{\mathrm{SPSR}}\left(C_{\mathrm{th}}\right)=1+\sum_{k=1}^{M}{\left(-1\right)}^{k}C_{M}^{k}\exp\left(-\lambda_{\mathrm{RD}}\xi -k\lambda_{\mathrm{SR}}\vartheta \right)\\ \; & +\sum_{k=1}^{M}\sum_{m=1}^{N}\frac{\pi {\left(-1\right)}^{k}C_{M}^{k}\lambda_{\mathrm{RD}}\xi }{2N}\sqrt{1-\mu_{m}^{2}}\exp\left(-\frac{\lambda_{\mathrm{RD}}\xi }{2}-\frac{k\lambda_{\mathrm{SR}}\gamma_{th}}{\eta \rho \Psi \Xi (\theta_{m})}-\frac{\lambda_{\mathrm{RD}}\xi \theta_{m}}{2}\right). \end{array}$$

where $\xi ,\vartheta ,\mu_{m},\theta_{m},N$ are given in (15). Q.E.D.  □

#### 3.1.2. Intercept Probability Analysis

IP is defined as the probability that the eavesdropper is able to wiretap the confidential information from S_*n*_ to D via R. The IP under the SPSR scheme is given in (19) at the top of the next page.

(19) $$\begin{array}{cc} IP_{\mathrm{SPSR}}\left(C_{\mathrm{th}}\right) & =\frac{\lambda_{\mathrm{JE}}\Psi }{\lambda_{\mathrm{SE}}\gamma_{th}\Phi +\lambda_{\mathrm{JE}}\Psi }-\sum_{k=1}^{M}{\left(-1\right)}^{k}C_{M}^{k}\\ \; & \times \exp\left(\frac{k\lambda_{\mathrm{SR}}\lambda_{\mathrm{RE}}\gamma_{th}\Phi }{2\eta \rho \Psi \lambda_{\mathrm{JE}}}\right)W_{-1,\frac{1}{2}}\left(\frac{k\lambda_{\mathrm{SR}}\lambda_{\mathrm{RE}}\gamma_{th}\Phi }{\eta \rho \Psi \lambda_{\mathrm{JE}}}\right)\\ \; & +\sum_{k=1}^{M}\frac{{\left(-1\right)}^{k}C_{M}^{k}\lambda_{\mathrm{JE}}}{{\tilde{\lambda }}_{\mathrm{JE}}}\times \exp\left(\frac{k\lambda_{\mathrm{SR}}\lambda_{\mathrm{RE}}\gamma_{th}\Phi }{2\eta \rho \Psi {\tilde{\lambda }}_{\mathrm{JE}}}\right)\times W_{-1,\frac{1}{2}}\left(\frac{k\lambda_{\mathrm{SR}}\lambda_{\mathrm{RE}}\gamma_{th}\Phi }{\eta \rho \Psi {\tilde{\lambda }}_{\mathrm{JE}}}\right). \end{array}$$

Here, ${\tilde{\lambda }}_{\mathrm{JE}}=\frac{\lambda_{\mathrm{SE}}\gamma_{th}\Phi }{\Psi }+\lambda_{\mathrm{JE}}$ and $W\left(•\right)$ is the Whittaker function ([37], 9.220).

**Proof.**  Let us first formulate the IP as follows [38]:

(20) $$\begin{array}{cc} \; & {\mathrm{IP}}_{\mathrm{SPSR}}\left(C_{\mathrm{th}}\right)=\mathrm{Pr}\left(C_{E}\geq C_{\mathrm{th}}\right)=\mathrm{Pr}\left(\gamma_{E}^{e2e}\geq \gamma_{th}\right),\\ \; & =1-\mathrm{Pr}\left(\max\left(\frac{\gamma_{S_{n}E}\Psi }{\Phi \gamma_{\mathrm{JE}}},\frac{\eta \rho \Psi \gamma_{S_{n}R}\gamma_{\mathrm{RE}}}{\Phi \gamma_{\mathrm{JE}}}\right)<\gamma_{th}\right)\\ \; & =1-\int_{0}^{\infty }Q\left(x\right)f_{\gamma_{\mathrm{JE}}}\left(x\right)dx, \end{array}$$

where $Q\left(x\right)$ is defined as follows:

(21) $$\begin{array}{cc} \; & Q\left(x\right)\;=\;\mathrm{Pr}\;\left(\left.\max\left(\frac{\gamma_{S_{n}E}\Psi }{\Phi x},\frac{\eta \rho \Psi \gamma_{S_{n}R}\gamma_{\mathrm{RE}}}{\Phi x}\right)\;<\gamma_{\mathrm{th}}\;\right|\;x\;=\;\gamma_{\mathrm{JE}}\;\right)\\ \; & =\underset{Q_{1}\left(x\right)}{\underset{︸}{\mathrm{Pr}\left(\frac{\gamma_{S_{n}E}\Psi }{\Phi x}<\gamma_{th}\right)}}\underset{Q_{2}\left(x\;\right)}{\underset{︸}{\mathrm{Pr}\left(\frac{\eta \rho \Psi \gamma_{S_{n}R}\gamma_{\mathrm{RE}}}{\Phi x}<\;\gamma_{th}\;\right)}}\;. \end{array}$$

The last equation in (21) is held due to the independence of the direct and indirect link from S to E via R. $Q_{1}\left(x\right)$ and $Q_{2}\left(x\right)$ are computed as follows:

(22) $$\begin{array}{cc} Q_{1}\left(x\right)= & \mathrm{Pr}\left(\frac{\gamma_{S_{n}E}\Psi }{\Phi x}<\gamma_{th}\right)=\mathrm{Pr}\left(\gamma_{S_{n}E}<\frac{\gamma_{th}\Phi x}{\Psi }\right)=1-\exp\left(-\frac{\lambda_{\mathrm{SE}}\gamma_{th}\Phi x}{\Psi }\right),\\ Q_{2}\left(x\right)= & \mathrm{Pr}\left(\frac{\eta \rho \Psi \gamma_{S_{n}R}\gamma_{\mathrm{RE}}}{\Phi x}<\gamma_{th}\right)=\int_{y=0}^{\infty }F_{\gamma_{S_{n}R}}\left(\frac{\gamma_{th}\Phi x}{\eta \rho \Psi y}\right)f_{\gamma_{\mathrm{RE}}}\left(y\right)dy\\ \overset{\left(a\right)}{=} & 1+\sum_{k=1}^{M}{\left(-1\right)}^{k}C_{M}^{k}\int_{0}^{\infty }\lambda_{\mathrm{RE}}\exp\left(-\frac{k\lambda_{\mathrm{SR}}\gamma_{th}\Phi x}{\eta \rho \Psi y}-\lambda_{\mathrm{RE}}y\right)dy \end{array}$$

(23) $$\begin{array}{cc} \overset{\left(b\right)}{=} & 1+2\sum_{k=1}^{M}{\left(-1\right)}^{k}C_{M}^{k}\sqrt{\frac{k\lambda_{\mathrm{SR}}\lambda_{\mathrm{RE}}\gamma_{th}\Phi x}{\eta \rho \Psi }}K_{1}\left(2\sqrt{\frac{k\lambda_{\mathrm{SR}}\lambda_{\mathrm{RE}}\gamma_{th}\Phi x}{\eta \rho \Psi }}\right), \end{array}$$

where $\left(a\right)$ is obtained with the assistance of Lemma 1; $\left(b\right)$ is held with the help of ([37], 3.324.1). $K_{v}\left(•\right)$ is the modified Bessel function of the second kind and *v*-th order.

By substituting (22) and (23) into (21), $Q\left(x\right)$ can be rewritten as follows:

(24) $$\begin{array}{cc} 1-Q\left(x\right)= & \Upsilon_{1}\left(x\right)-\Upsilon_{2}\left(x\right)+\Upsilon_{3}\left(x\right)\\ \Upsilon_{1}\left(x\right)= & \exp\left(-\frac{\lambda_{\mathrm{SE}}\gamma_{th}\Phi x}{\Psi }\right)\\ \Upsilon_{2}\left(x\right)= & 2\sum_{k=1}^{M}{\left(-1\right)}^{k}C_{M}^{k}\sqrt{\frac{k\lambda_{\mathrm{SR}}\lambda_{\mathrm{RE}}\gamma_{th}\Phi x}{\eta \rho \Psi }}K_{1}\left(2\sqrt{\frac{k\lambda_{\mathrm{SR}}\lambda_{\mathrm{RE}}\gamma_{th}\Phi x}{\eta \rho \Psi }}\right), \end{array}$$

(25) $$\begin{array}{cc} \; & \Upsilon_{3}\left(x\right)=2\sum_{k=1}^{M}{\left(-1\right)}^{k}C_{M}^{k}\exp\left(-\frac{\lambda_{\mathrm{SE}}\gamma_{th}\Phi x}{\Psi }\right)\sqrt{\frac{k\lambda_{\mathrm{SR}}\lambda_{\mathrm{RE}}\gamma_{th}\Phi x}{\eta \rho \Psi }}K_{1}\left(2\sqrt{\frac{k\lambda_{\mathrm{SR}}\lambda_{\mathrm{RE}}\gamma_{th}\Phi x}{\eta \rho \Psi }}\right), \end{array}$$

Substituting (24) and (25) into (20), ${\mathrm{IP}}_{\mathrm{SPSR}}$ is then rewritten as

(26) $$\begin{array}{cc} {\mathrm{IP}}_{\mathrm{SPSR}}\left(C_{\mathrm{th}}\right)= & \int_{0}^{\infty }\left(\Upsilon_{1}\left(x\right)-\Upsilon_{2}\left(x\right)+\Upsilon_{3}\left(x\right)\right)f_{\gamma_{\mathrm{JE}}}\left(x\right)dx\\ = & \underset{I_{1}}{\underset{︸}{\int_{0}^{\infty }\Upsilon_{1}\left(x\right)f_{\gamma_{\mathrm{JE}}}\left(x\right)dx}}-\underset{I_{2}}{\underset{︸}{\int_{0}^{\infty }\Upsilon_{2}\left(x\right)f_{\gamma_{\mathrm{JE}}}\left(x\right)dx}}+\underset{I_{3}}{\underset{︸}{\int_{0}^{\infty }\Upsilon_{3}\left(x\right)f_{\gamma_{\mathrm{JE}}}\left(x\right)dx}}, \end{array}$$

where

(27) $$\begin{array}{cc} I_{1}= & \int_{0}^{\infty }\lambda_{\mathrm{JE}}\exp\left(-\frac{\lambda_{\mathrm{SE}}\gamma_{th}\Phi x}{\Psi }-\lambda_{\mathrm{JE}}x\right)dx\\ = & \lambda_{\mathrm{JE}}\int_{0}^{\infty }\exp\left(-x\left(\frac{\lambda_{\mathrm{SE}}\gamma_{th}\Phi }{\Psi }+\lambda_{\mathrm{JE}}\right)\right)dx=\frac{\lambda_{\mathrm{JE}}\Psi }{\lambda_{\mathrm{SE}}\gamma_{th}\Phi +\lambda_{\mathrm{JE}}\Psi }, \end{array}$$

(28) $$\begin{array}{cc} I_{2}= & 2\sum_{k=1}^{M}{\left(-1\right)}^{k}C_{M}^{k}\lambda_{\mathrm{JE}}\int_{0}^{\infty }\sqrt{\frac{k\lambda_{\mathrm{SR}}\lambda_{\mathrm{RE}}\gamma_{th}\Phi x}{\eta \rho \Psi }}\exp\left(-\lambda_{\mathrm{JE}}x\right)K_{1}\left(2\sqrt{\frac{k\lambda_{\mathrm{SR}}\lambda_{\mathrm{RE}}\gamma_{th}\Phi x}{\eta \rho \Psi }}\right)dx,\\ \; & \overset{\left(a\right)}{=}\sum_{k=1}^{M}{\left(-1\right)}^{k}C_{M}^{k}\exp\left(\frac{k\lambda_{\mathrm{SR}}\lambda_{\mathrm{RE}}\gamma_{th}\Phi }{2\eta \rho \Psi \lambda_{\mathrm{JE}}}\right)W_{-1,\frac{1}{2}}\left(\frac{k\lambda_{\mathrm{SR}}\lambda_{\mathrm{RE}}\gamma_{th}\Phi }{\eta \rho \Psi \lambda_{\mathrm{JE}}}\right),\\ I_{3}= & 2\sum_{k=1}^{M}{\left(-1\right)}^{k}C_{M}^{k}\lambda_{\mathrm{JE}}\int_{0}^{\infty }\exp\left(-x\left[\frac{\lambda_{\mathrm{SE}}\gamma_{th}\Phi }{\Psi }+\lambda_{\mathrm{JE}}\right]\right)\\ \; & \times \sqrt{\frac{k\lambda_{\mathrm{SR}}\lambda_{\mathrm{RE}}\gamma_{th}\Phi x}{\eta \rho \Psi }}K_{1}\left(2\sqrt{\frac{k\lambda_{\mathrm{SR}}\lambda_{\mathrm{RE}}\gamma_{th}\Phi x}{\eta \rho \Psi }}\right)dx,\\ = & \sum_{k=1}^{M}\frac{{\left(-1\right)}^{k}C_{M}^{k}\lambda_{\mathrm{JE}}}{{\tilde{\lambda }}_{\mathrm{JE}}}\exp\left(\frac{k\lambda_{\mathrm{SR}}\lambda_{\mathrm{RE}}\gamma_{th}\Phi }{2\eta \rho \Psi {\tilde{\lambda }}_{\mathrm{JE}}}\right)W_{-1,\frac{1}{2}}\left(\frac{k\lambda_{\mathrm{SR}}\lambda_{\mathrm{RE}}\gamma_{th}\Phi }{\eta \rho \Psi {\tilde{\lambda }}_{\mathrm{JE}}}\right), \end{array}$$

where ${\tilde{\lambda }}_{\mathrm{JE}}=\frac{\lambda_{\mathrm{SE}}\gamma_{th}\Phi }{\Psi }+\lambda_{\mathrm{JE}}$, $\left(a\right)$ is attained by yielding ([37], 6.643.3).

Finally, by substituting (27) and (28) into (26), we obtain the $IP_{\mathrm{SPSR}}$ and close the proof here.  □

### 3.2. Dynamic Power Splitting-Based Relaying (DPSR)

Under the DPSR scheme, the PS ratio denoted by $\rho^{*}$ is properly turned so that the e2e SNR at D is maximized, thereby maximizing the system capacity. As the DF protocol is taken into consideration, $\rho^{*}$ can be derived as follows:

(29) $$\begin{array}{c} \gamma_{R}=\gamma_{D}↔\left(1-\rho^{*}\right)\gamma_{S_{n}R}\Psi =\eta \rho^{*}\Psi \gamma_{S_{n}R}\gamma_{\mathrm{RD}}\\ \;\to \rho^{*}=\frac{1}{\eta \gamma_{\mathrm{RD}}+1}\in \left(0,1\right). \end{array}$$

Through direct inspection (29), we observe that $\rho^{*}$ relies only on the instantaneous CSI of the second hop from R to D and is different from the literature where $\rho^{*}$ is a high complexity function of the channel gain, the targeted rate and the transmit power of the source node [36].

#### 3.2.1. OP Analysis

The OP under the DPSR protocol is evaluated as follows:

(30) $$\begin{array}{cc} \; & {\mathrm{OP}}_{\mathrm{DPSR}}\left(C_{\mathrm{th}}\right)=1+2\sum_{k=1}^{M}{\left(-1\right)}^{k}C_{M}^{k}\exp\left(-\frac{k\lambda_{\mathrm{SR}}\gamma_{th}}{\Psi }\right)\sqrt{\frac{k\lambda_{\mathrm{SR}}\lambda_{\mathrm{RD}}\gamma_{th}}{\eta \Psi }}K_{1}\left(2\sqrt{\frac{k\lambda_{\mathrm{SR}}\lambda_{\mathrm{RD}}\gamma_{th}}{\eta \Psi }}\right). \end{array}$$

**Proof.**  Let us start the proof with the definition of ${\mathrm{OP}}_{\mathrm{DPSR}}$ as follows:

$$\begin{array}{cc} \; & {\mathrm{OP}}_{\mathrm{DPSR}}\left(\;C_{\mathrm{th}}\;\right)\;=\;\mathrm{Pr}\left(\gamma_{D}<\gamma_{\mathrm{th}}\right)=\mathrm{Pr}\left(\frac{\eta \Psi \gamma_{S_{n}R}\gamma_{\mathrm{RD}}}{\eta \gamma_{\mathrm{RD}}+1}\;<\;\gamma_{th}\;\right)\\ \; & =\mathrm{Pr}\left(\gamma_{S_{n}R}<\frac{\gamma_{th}\left(\eta \gamma_{\mathrm{RD}}+1\right)}{\eta \Phi \gamma_{\mathrm{RD}}}\right)=\int_{0}^{\infty }F_{\gamma_{S_{n}R}}\left(\frac{\gamma_{th}\left(\eta x+1\right)}{\eta \Phi x}\right)f_{\gamma_{\mathrm{RD}}}\left(x\right)dx,\\ \; & \overset{\left(a\right)}{=}1+\sum_{k=1}^{M}{\left(-1\right)}^{k+1}C_{M}^{k}\exp\left(-\frac{k\lambda_{\mathrm{SR}}\gamma_{th}}{\Psi }\right)\int_{0}^{\infty }\lambda_{\mathrm{RD}}\exp\left(-\frac{k\lambda_{\mathrm{SR}}\gamma_{th}}{\eta \Psi x}-\lambda_{\mathrm{RD}}x\right)dx,\\ \; & =1+2\sum_{k=1}^{M}{\left(-1\right)}^{k}C_{M}^{k}\exp\left(-\frac{k\lambda_{\mathrm{SR}}\gamma_{th}}{\Psi }\right)\sqrt{\frac{k\lambda_{\mathrm{SR}}\lambda_{\mathrm{RD}}\gamma_{th}}{\eta \Psi }}K_{1}\left(2\sqrt{\frac{k\lambda_{\mathrm{SR}}\lambda_{\mathrm{RD}}\gamma_{th}}{\eta \Psi }}\right), \end{array}$$

where $\left(a\right)$ is derived with the help of Lemma 1. Finally, (30) is obtained by using ([37], 3.324.1).  □

#### 3.2.2. IP Analysis

In this section, we derive the IP of the eavesdropper under the DPSR scheme. Particularly, ${\mathrm{IP}}_{\mathrm{DPSR}}$ is computed by (31) at the top of the next page.

(31) $$\begin{array}{cc} {\mathrm{IP}}_{\mathrm{DPSR}}\left(C_{\mathrm{th}}\right)= & \frac{\lambda_{\mathrm{JE}}\Psi }{\lambda_{\mathrm{JE}}\Psi +\lambda_{\mathrm{SE}}\gamma_{th}\Phi }-\sum_{t=0}^{\infty }{\sum_{k=1}^{M}{\left(-1\right)}^{t+k}C_{M}^{k}\left(\frac{\lambda_{\mathrm{RD}}}{\eta }\right)}^{t+1}\\ \; & \times \left[G_{2,3}^{3,1}\left(\frac{\zeta_{1}\left(k\right)}{\lambda_{\mathrm{JE}}}\left|\begin{array}{c} 0,0\\ -t-1,1,0 \end{array}\right.\right)-\frac{\lambda_{\mathrm{JE}}}{{\tilde{\lambda }}_{\mathrm{JE}}}G_{2,3}^{3,1}\left(\frac{\zeta_{1}\left(k\right)}{{\tilde{\lambda }}_{\mathrm{JE}}}\left|\begin{array}{c} 0,0\\ -t-1,1,0 \end{array}\right.\right)\right] \end{array}$$

$\zeta_{1}\left(k\right)=\frac{k\lambda_{\mathrm{SR}}\lambda_{\mathrm{RE}}\gamma_{th}\Phi }{\eta \Psi }$.

**Proof.**  Let us commence with the definition of IP as follows:

(32) $$\begin{array}{cc} \; & \;{\mathrm{IP}}_{\mathrm{DPSR}}\;\left(\;C_{\mathrm{th}}\;\right)=\;\mathrm{Pr}\left(\max\left(\;\frac{\gamma_{S_{n}E}\Psi }{\Phi \gamma_{\mathrm{JE}}}\;,\;\frac{\eta \Psi \gamma_{S_{n}R}\gamma_{\mathrm{RE}}}{\Phi \gamma_{\mathrm{JE}}\left(\eta \gamma_{\mathrm{RD}}+1\right)}\right)\;\;\geq \;\;\gamma_{th}\;\right)\\ \; & =1-\int_{0}^{\infty }\underset{\hat{Q}\left(x\right)}{\underset{︸}{\mathrm{Pr}\left(\max\left(\gamma_{S_{n}E}\Psi ,\frac{\eta \Psi \gamma_{S_{n}R}\gamma_{\mathrm{RE}}}{\eta \gamma_{\mathrm{RD}}+1}\right)\;<\;\gamma_{th}\Phi x\;\right)}}f_{\gamma_{\mathrm{JE}}}\left(x\right)dx, \end{array}$$

where

(33) $$\begin{array}{cc} \hat{Q}\left(x\right)= & \mathrm{Pr}\left(\max\left(\gamma_{S_{n}E}\Psi ,\frac{\eta \Psi \gamma_{S_{n}R}\gamma_{\mathrm{RE}}}{\eta y+1}\right)<\gamma_{th}\Phi x\right)\\ = & \int_{y=0}^{\infty }\underset{{\overset{⌢}{\;Q}}_{2}\left(x,y\right)}{\underset{︸}{\mathrm{Pr}\left(\frac{\eta \Psi \gamma_{S_{n}R}\gamma_{\mathrm{RE}}}{\eta y+1}<\gamma_{th}\Phi x\right)}}\underset{{\overset{⌢}{\;Q}}_{1}\left(x\right)}{\underset{︸}{\mathrm{Pr}\left(\gamma_{S_{n}E}\Psi <\gamma_{th}\Phi x\right)}}f_{\gamma_{\mathrm{RD}}}\left(y\right)dy \end{array}$$

where ${\hat{Q}}_{1}\left(x\right)$ and ${\overset{⌢}{\;Q}}_{2}$ (*x*,*y*) are given as follows:

$$\begin{array}{l} {\hat{Q}}_{1}\left(x\right)=1-\exp\left(-\frac{\lambda_{\mathrm{SE}}\gamma_{th}\Phi x}{\Psi }\right),\\ {\overset{⌢}{\;Q}}_{2}\left(x,y\right)=1+2\sum_{k=1}^{M}{\left(-1\right)}^{k}C_{M}^{k}\sqrt{\frac{k\lambda_{\mathrm{SR}}\lambda_{\mathrm{RE}}\gamma_{th}\Phi }{\eta \Psi }}\sqrt{x\left(\eta y+1\right)}K_{1}\left(2\sqrt{\frac{k\lambda_{\mathrm{SR}}\lambda_{\mathrm{RE}}\gamma_{th}\Phi x\left(\eta y+1\right)}{\eta \Psi }}\right). \end{array}$$

The IP in (32) is then computed as follows:

(34) $$\begin{array}{cc} \hat{Q}\left(x\right)= & 1-\;\int_{y=0}^{\infty }{\hat{Q}}_{1}\left(x\right){\hat{Q}}_{2}\left(x,y\right)f_{\gamma_{\mathrm{RD}}}\left(y\right)\;dy,={\hat{I}}_{1}\left(x\right)-{\hat{I}}_{2}\left(x\right)+{\hat{I}}_{3}\left(x\right), \end{array}$$

where

(35) $$\begin{array}{cc} \; & {\hat{I}}_{1}\left(x\right)=\exp\left(-\frac{\lambda_{\mathrm{SE}}\gamma_{th}\Phi x}{\Psi }\right)\\ \; & {\hat{I}}_{2}\left(x\right)=2\sum_{k=1}^{M}{\left(-1\right)}^{k}C_{M}^{k}\lambda_{\mathrm{RD}}\int_{0}^{\infty }\exp\left(-\lambda_{\mathrm{RD}}y\right)\sqrt{\zeta_{1}\left(k\right)x\left(\eta y+1\right)}K_{1}\left(2\sqrt{\zeta_{1}\left(k\right)x\left(\eta y+1\right)}\right)dy\\ \; & \overset{\left(a\right)}{=}2\sum_{t=0}^{\infty }\sum_{k=1}^{M}\frac{{\left(-1\right)}^{t+k}C_{M}^{k}{\left(\lambda_{\mathrm{RD}}\right)}^{t+1}}{t!}\int_{0}^{\infty }y^{t}\sqrt{\zeta_{1}\left(k\right)x\left(\eta y+1\right)}K_{1}\left(2\sqrt{\zeta_{1}\left(k\right)x\left(\eta y+1\right)}\right)dy\\ \; & \overset{\left(b\right)}{=}\sum_{t=0}^{\infty }{\sum_{k=1}^{M}{\left(-1\right)}^{t+k}C_{M}^{k}\left(\frac{\lambda_{\mathrm{RD}}}{\eta }\right)}^{t+1}G_{1,3}^{3,0}\left(\zeta_{1}\left(k\right)x\left|\begin{array}{c} 0\\ -t-1,1,0 \end{array}\right.\right), \end{array}$$

where $\left(a\right)$ is obtained by representing the exponential function in the infinity series form, i.e., $\exp\left(-\lambda_{\mathrm{RD}}y\right)=\sum_{t=0}^{\infty }\frac{{\left(-\lambda_{\mathrm{RD}}y\right)}^{t}}{t!}=\sum_{t=0}^{\infty }\frac{{\left(-1\right)}^{t}{\left(\lambda_{\mathrm{RD}}\right)}^{t}}{t!}y^{t}$ and $\left(b\right)$ is held with the help of ([37], 6.592.4); $\zeta_{1}\left(k\right)=\frac{k\lambda_{\mathrm{SR}}\lambda_{\mathrm{RE}}\gamma_{th}\Phi }{\eta \Psi }$ and $G_{p,q}^{m,n}\left(z\left|\begin{array}{c} a_{1},\ldots ,a_{p}\\ b_{1},\ldots ,b_{q} \end{array}\right.\right)$ is the Meijer-G function.

$$\begin{array}{cc} \; & {\hat{I}}_{3}\left(x\right)=2\sum_{k=1}^{M}{\left(-1\right)}^{k}C_{M}^{k}\lambda_{\mathrm{RD}}\exp\left(-\frac{\lambda_{\mathrm{SE}}\gamma_{th}\Phi x}{\Psi }\right)\\ \; & \times \int_{0}^{\infty }\exp\left(-\lambda_{\mathrm{RD}}y\right)\sqrt{\zeta_{1}\left(k\right)x\left(\eta y+1\right)}K_{1}\left(2\sqrt{\zeta_{1}\left(k\right)x\left(\eta y+1\right)}\right)dy\\ \; & =\sum_{t=0}^{\infty }{\sum_{k=1}^{M}{\left(-1\right)}^{t+k}C_{M}^{k}\left(\frac{\lambda_{\mathrm{RD}}}{\eta }\right)}^{t+1}\exp\left(-\frac{\lambda_{\mathrm{SE}}\gamma_{th}\Phi x}{\Psi }\right)G_{1,3}^{3,0}\left(\zeta_{1}\left(k\right)x\left|\begin{array}{c} 0\\ -t-1,1,0 \end{array}\right.\right). \end{array}$$

Finally, substituting (35) and (36) into (32), we have

(36) $$\begin{array}{cc} \; & {\mathrm{IP}}_{\mathrm{SPSR}}=\Xi_{1}-\Xi_{2}+\Xi_{3}\\ \; & \Xi_{1}=\lambda_{\mathrm{JE}}\int_{0}^{\infty }\exp\left(-\frac{\lambda_{\mathrm{SE}}\gamma_{th}\Phi x}{\Psi }-\lambda_{\mathrm{JE}}x\right)dx=\frac{\lambda_{\mathrm{JE}}\Psi }{\lambda_{\mathrm{JE}}\Psi +\lambda_{\mathrm{SE}}\gamma_{th}\Phi }\\ \; & \Xi_{2}=\sum_{t=0}^{\infty }{\sum_{k=1}^{M}{\left(-1\right)}^{t+k}C_{M}^{k}\lambda_{\mathrm{JE}}\left(\frac{\lambda_{\mathrm{RD}}}{\eta }\right)}^{t+1}\int_{0}^{\infty }\exp\left(-\lambda_{\mathrm{JE}}x\right)G_{1,3}^{3,0}\left(\zeta_{1}\left(k\right)x\left|\begin{array}{c} 0\\ -t-1,1,0 \end{array}\right.\right)dx\\ \; & \overset{\left(a\right)}{=}\sum_{t=0}^{\infty }{\sum_{k=1}^{M}{\left(-1\right)}^{t+k}C_{M}^{k}\left(\frac{\lambda_{\mathrm{RD}}}{\eta }\right)}^{t+1}G_{2,3}^{3,1}\left(\frac{\zeta_{1}\left(k\right)}{\lambda_{\mathrm{JE}}}\left|\begin{array}{c} 0,0\\ -t-1,1,0 \end{array}\right.\right) \end{array}$$

(37) $$\begin{array}{cc} \Xi_{3}= & \sum_{t=0}^{\infty }{\sum_{k=1}^{M}{\left(-1\right)}^{t+k}C_{M}^{k}\lambda_{\mathrm{JE}}\left(\frac{\lambda_{\mathrm{RD}}}{\eta }\right)}^{t+1}\\ \; & \times \int_{0}^{\infty }\exp\left(-x\left[\frac{\lambda_{\mathrm{SE}}\gamma_{th}\Phi }{\Psi }+\lambda_{\mathrm{JE}}\right]\right)G_{1,3}^{3,0}\left(\zeta_{1}\left(k\right)x\left|\begin{array}{c} 0\\ -t-1,1,0 \end{array}\right.\right)dx\\ = & \sum_{t=0}^{\infty }{\sum_{k=1}^{M}{\left(-1\right)}^{t+k}C_{M}^{k}\left(\frac{\lambda_{\mathrm{RD}}}{\eta }\right)}^{t+1}\frac{\lambda_{\mathrm{JE}}}{{\tilde{\lambda }}_{\mathrm{JE}}}G_{2,3}^{3,1}\left(\frac{\zeta_{1}\left(k\right)}{{\tilde{\lambda }}_{\mathrm{JE}}}\left|\begin{array}{c} 0,0\\ -t-1,1,0 \end{array}\right.\right). \end{array}$$

where $\left(a\right)$ is achieved by borrowing the results from ([37], 7.813.1) and $\Xi_{3}$ is given at the top of the next page. Q.E.D.  □

## 4. Numerical Results

In this section, we provide numerical results to not only verify the accuracy of the proposed mathematical frameworks but also discuss the behaviors of the considered systems under the impact of various important parameters. Unless otherwise stated, the following parameters are utilized: $C_{\mathrm{th}}$ = 0.5 bps/Hz, η = 0.8, Ψ = 5 dB, Φ = 1 dB, M = 3 and β = 2.5. For clarity, simulation parameters are listed in Table 2. Simulation results are obtained by the Monte Carlo method [39] and are averaged over $10^{6}$ channel realizations.

**Figure 5:**
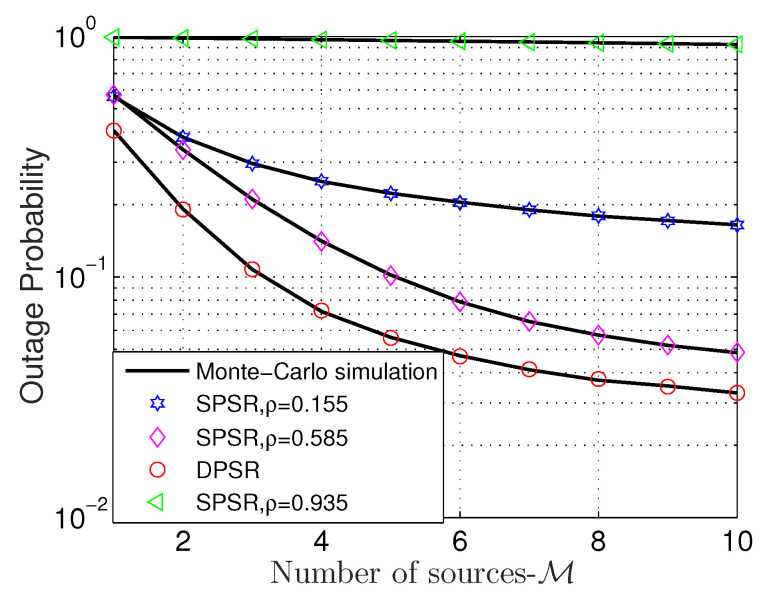
OP versus M with $C_{\mathrm{th}}$ = 0.5 (bps/Hz), η = 0.8, and Φ = 1 dB. Markers are plotted from (19) and (31), respectively.

**Figure 6:**
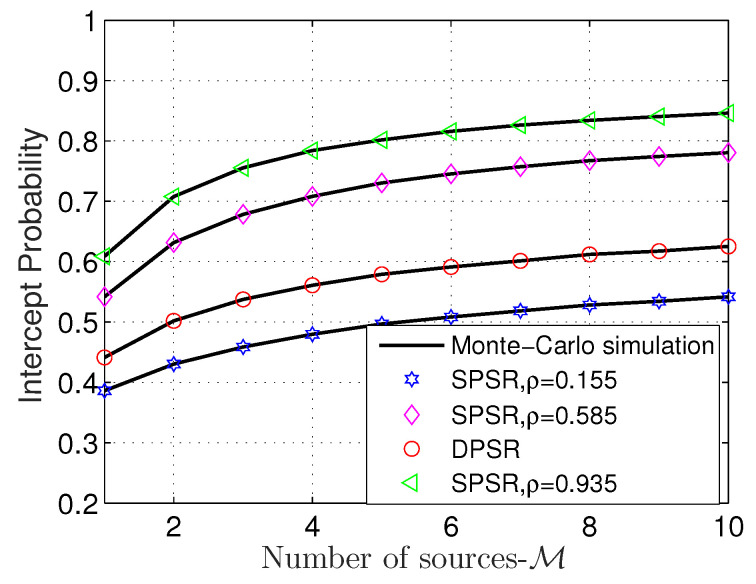
OP versus M with $C_{\mathrm{th}}$ = 0.5 (bps/Hz), η = 0.8, and Φ = 1 dB.

Figure 5 and Figure 6 unveil the behavior of both OP and IP regarding the number of source M. From Figure 5, there is no doubt that raising M enhances the OP’s performance. However, there is a difference from the pace of improvement when M is small and large. More precisely, OP dramatically decreases when M goes from 1 to 5; it then slightly reduces from this point. Furthermore, Figure 5 also shows that the DPSR scheme is far better than its counterpart. Particularly, the DPSR protocol is better than the SPSR 5× with ρ=0.155 and over 10× with ρ=0.935 when M=10. Concerning the SPSR scheme only, we see that ρ=0.585 outperforms others, i.e., ρ=0.155 and ρ=0.935.

Figure 6 shows the IP performance versus M. We first see that the DPSR scheme is neither the best nor the worst scheme under the current setup. According to this figure, the SPSR with ρ=0.935 is the best one followed by SPSR with ρ=0.585, DPSR and SPSR with ρ=0.155. Next, similar to OP, increasing M improves the IP as well; thus, there is a higher probability that the confidential information from S to D is wiretapped by E. Figure 5 and Figure 6 illustrate that the performance of OP and IP is contradictory.

**Figure 7:**
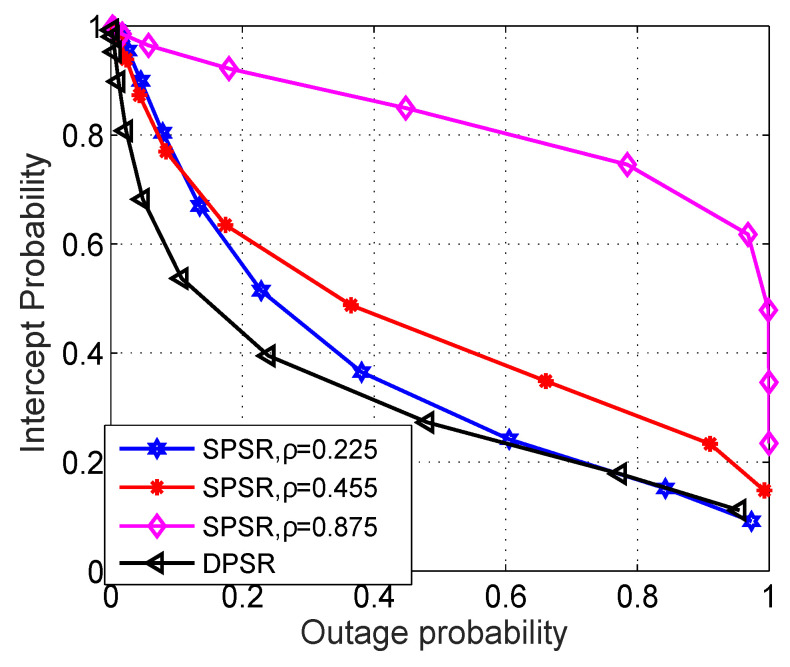
OP versus IP with $C_{\mathrm{th}}$ = 0.5 (bps/Hz), η = 0.8, and M = 3. Markers are plotted from (15) and (30), respectively.

In Figure 7, we study the interaction between OP and IP. Generally, the DPSR scheme is superior to the SPSR, since it has the smallest area covered by the curve and the horizontal axis. Moreover, the larger the ρ and the higher the OP and IP, the worse the system performance. The main reason is that although the received power at the energy harvester of R is directly proportional to ρ, the received power at the information decoder, however, is inversely proportional to ρ; hence, OP is becoming worse. On the other hand, the SNR at E is scaling up with $P_{R}$; hence, IP keeps increasing. Moreover, this figure also reveals that increasing OP simply declines the IP. This can be explained by the OP going up, meaning that the transmission between S and D is probably dropped out, thus decreasing the IP.

**Figure 8:**
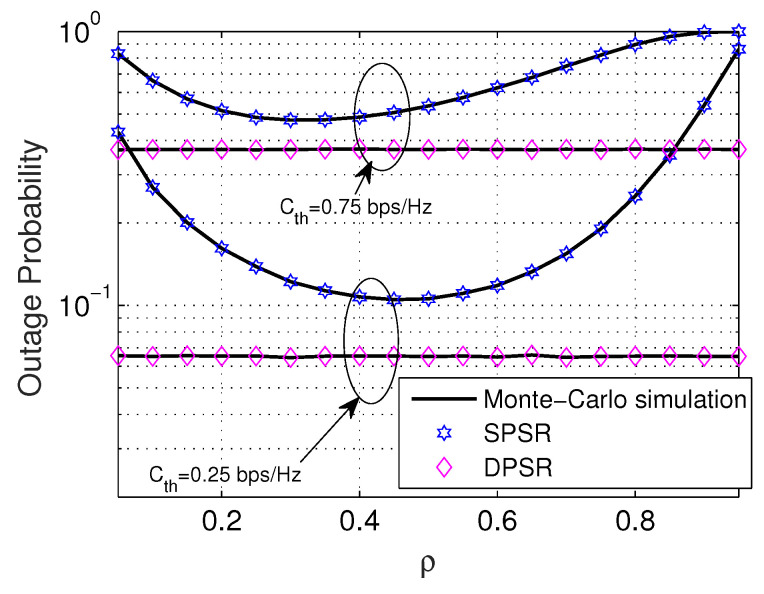
OP versus ρ with $C_{\mathrm{th}}$ = 0.5 (bps/Hz), η = 0.8, M = 3, and Ψ = 5 dB.

**Figure 9:**
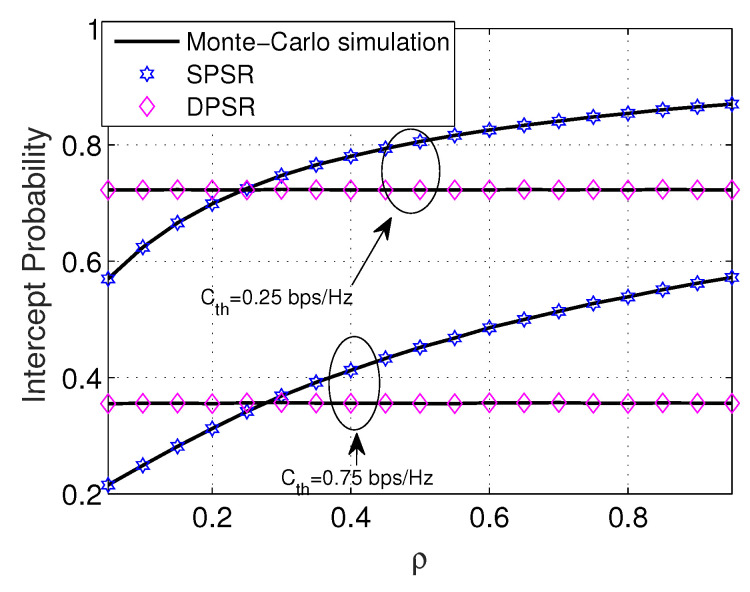
IP versus ρ with $C_{\mathrm{th}}$ = 0.5 (bps/Hz), η = 0.8, Ψ = 5 dB, M = 3, and Φ = 1 dB.

Figure 8 and Figure 9 investigate the trend of OP and IP versus the power-splitting ratio ρ. In Figure 8, we see that the DPSR scheme is independent of ρ, since $\rho^{*}$ counts only on the channel gain from R to D. Furthermore, the DPSR scheme is always superior to the SPSR regardless of the value of ρ. Regarding the SPSR scheme, it is a convex function of ρ. Particularly, the SPSR scheme starts decreasing until its peak; then, it turns over and keeps increasing to one when ρ goes from zero to one. It is certain that the smaller the $C_{\mathrm{th}}$, the better the OP. Figure 9 stretches the impact of the PS ratio ρ on the performance of the IP. Despite DPSR having the same behavior as the OP, the SPSR scheme experiences differently. Particularly, it is a monotonic increasing function of ρ. The main reason is that the e2e SNR of E in (11) is an increasing function of ρ. This is the outcome of the increase in ρ: that IP is a monotonic increasing function with respect to ρ. Nevertheless, different from OP, the DPSR scheme is not constantly larger than that of the SPSR scheme.

**Figure 10:**
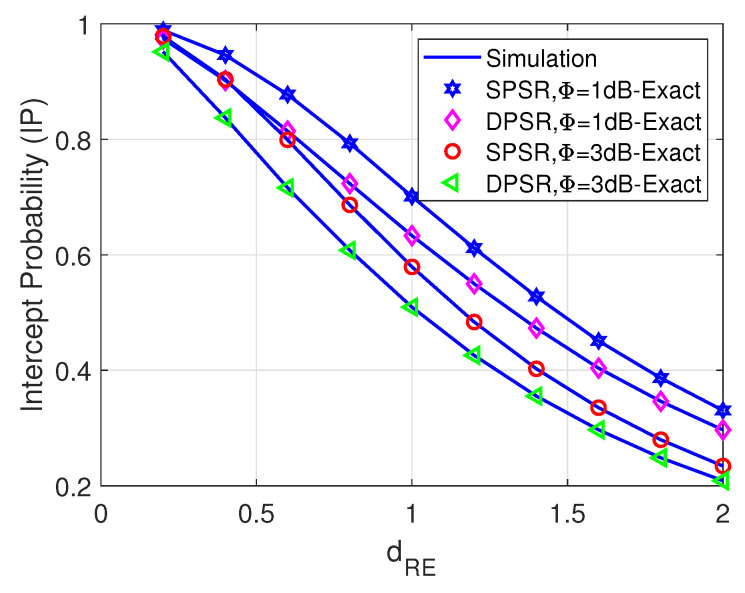
IP versus $d_{\mathrm{RE}}$ with M = 2, η = 0.8, Ψ = 5 dB, ρ = 0.5 and $d_{\mathrm{SE}}=\sqrt{{\left(d_{S_{n}R}\right)}^{2}+{\left(d_{\mathrm{RE}}\right)}^{2}}$.

Figure 10 unveils the impact of the mobility of eavesdropper E on the performance of IP. Particularly, E moves along the perpendicular lines of $d_{S_{n}R}$ so that $d_{\mathrm{SE}}=\sqrt{{\left(d_{S_{n}R}\right)}^{2}+{\left(d_{\mathrm{RE}}\right)}^{2}}$ is always satisfied. It is evident that when E goes away from both S_*n*_ and R, the IP declines. This comes from the fact that the channel gain from both S_*n*_ and R to E reduces significantly due to the increase of the large-scale path loss. Furthermore, increasing the transmit power of jammer J improves the security of the considered networks, i.e., decreasing the IP.

## 5. Conclusions

The OP and IP were investigated in the present paper under the SWIPT-enabled relaying networks. Particularly, the OP and IP were derived under two distinguished schemes of the power-splitting ratio at the relay, i.e., the static and dynamic PS scheme. Our findings revealed that the OP of the DPSR scheme was generally better than another one for all values of ρ; however, this was not the case for the IP. We also found that the behavior of the OP and IP was contradicted with respect to the transmit power: the number of sources. The present work can be extended in various directions. One promising avenue involves integrating deep learning techniques to leverage a data-driven approach, thereby further optimizing the system’s performance [40]. A valuable aspect to explore is the comparison with covert communications [41,42]. Additionally, incorporating reconfigurable intelligent surfaces could provide a significant boost [43,44]. Further comprehensive investigations into the considered networks can be conducted by employing tools from stochastic geometry to capture the randomness inherent in wireless sensor networks [45,46]. Lastly, combining the proposed framework with Fountain codes has the potential to tremendously enhance both spectral and energy efficiency [47,48].

## Figures and Tables

**Table 1:** Main notations and mathematical symbols.

Notations	Descriptions
$P_{S_{n}}$	The transmit power of S_*n*_
$P_{R}$	The transmit power of R
$P_{J}$	The transmit power of J
$n_{X}$	The additive white Gaussian noise (AWGN) at node X∈R,D,E
$x_{X}$	The transmit signal of node $X\in R,J,S_{n}$
$\gamma_{X}$	The SNR at node X∈R,D
$\gamma_{E}^{X}$	The SNR at the eavesdropper E at the $X\in \left\{1,2\right\}$ phase
η	The energy conversion efficiency
ρ	The power-splitting ratio
${\mathrm{OP}}_{X}$	The outage probability of $X\in \left\{\mathrm{SPSR},\mathrm{DPSR}\right\}$ scheme
${\mathrm{IP}}_{X}$	The intercept probability of $X\in \left\{\mathrm{SPSR},\mathrm{DPSR}\right\}$ scheme
T	The time duration
β	The path-loss exponent
$d_{\mathrm{XY}}$	The transmission distance from node X to node Y
M	The number of source nodes
$\gamma_{\mathrm{XY}}={\left|h_{\mathrm{XY}}\right|}^{2}$	The channel gain from node X to node Y
$\mathrm{Pr}\left(•\right)$	The probability operator
$E\left\{•\right\}$	The expectation operator
$C_{\mathrm{th}}$	The targeted capacity
$\gamma_{th}=2^{2C_{\mathrm{th}}}-1$	The SNR threshold

**Table 2:** Simulation parameters.

Symbol	Parameter Name	Fixed Value	Varying Range
$C_{\mathrm{th}}$	Target rate	0.25; 0.5; 0.75 (bps/Hz)	none
η	EH efficiency	0.8	none
ρ	Power-splitting ratio	0.155; 0.225; 0.455; 0.585; 0.875; 0.935	0.05 to 0.95
$\lambda_{\mathrm{SR}}$	Parameter of $|h_{\mathrm{SR}}{|}^{2}$	1	none
$\lambda_{\mathrm{RD}}$	Parameter of $|h_{\mathrm{RD}}{|}^{2}$	0.5	none
$\lambda_{\mathrm{RE}}$	Parameter of $|h_{\mathrm{RE}}{|}^{2}$	1	0.0179 to 4
$\lambda_{\mathrm{SE}}$	Parameter of $|h_{\mathrm{RE}}{|}^{2}$	2	1 to 13.1188
$\lambda_{\mathrm{JE}}$	Parameter of $|h_{\mathrm{RD}}{|}^{2}$	1	none
β	Path-loss exponent	2.5	none
Ψ	Transmit power to noise ratio at source	5 dB	−5 to 20 (dB)
Φ	Transmit power to noise ratio at jammer	1; 3 dB	none
M	Number of sources	3	1 to 10

## Data Availability

The raw data supporting the conclusions of this article will be made available by the authors on request.

## Appendix A. Proof of Lemma 1

**Proof.** The CDF and PDF of the maximum of M i.i.d. exponential RVs with parameter λ are provided in this section. Let us start with the definition of the CDF as follows:

(A1) $$\begin{array}{cc} F_{X_{\max}}\left(x\right)= & \mathrm{Pr}\left(X_{\max}=\max_{m\in \left\{1,\ldots ,M\right\}}\left\{X_{m}\right\}<x\right)\\ \overset{\left(a\right)}{=} & \prod_{m=1}^{M}F_{X_{m}}\left(x\right)\overset{\left(b\right)}{=}{\left(1-\exp\left(-\lambda x\right)\right)}^{M}\\ \overset{\left(c\right)}{=} & 1+\sum_{m=1}^{M}{\left(-1\right)}^{m}C_{M}^{m}\exp\left(-m\lambda x\right), \end{array}$$

where $\left(a\right)$ is attained due to the independence between RVs. By substituting the CDF of $X_{m}$, we obtain $\left(b\right)$. Finally, by employing the binomial theorem, we attain $\left(c\right)$. The PDF is then derived immediately by taking the first-order derivative of CDF with respect to *x* and is given as

(A2) $$\begin{array}{cc} f_{X_{\max}}\left(x\right)= & \frac{\partial F_{X_{\max}}\left(x\right)}{\partial x}=\lambda \sum_{m=1}^{M-1}{\left(-1\right)}^{m}\\ \; & \times C_{M-1}^{m}\exp\left(-\left(m+1\right)\lambda x\right). \end{array}$$

We close the proof here.  □
